# Assessment of contemporary genetic diversity and inter-taxa/inter-region exchange of avian paramyxovirus serotype 1 in wild birds sampled in North America

**DOI:** 10.1186/s12985-017-0714-8

**Published:** 2017-03-03

**Authors:** Andrew M. Ramey, Iryna V. Goraichuk, Joseph T. Hicks, Kiril M. Dimitrov, Rebecca L. Poulson, David E. Stallknecht, Justin Bahl, Claudio L. Afonso

**Affiliations:** 1 US Geological Survey, Alaska Science Center, 4210 University Drive, Anchorage, Alaska, 99508 USA; 2 US Department of Agriculture, Agriculture Research Service, US National Poultry Research Center, Southeast Poultry Research Laboratory, 934 College Station Road, Athens, GA 30605 USA; 3 University of Texas, School of Public Health, 1200 Pressler Street, Houston, TX 77030 USA; 4 Department of Population Health, Southeastern Cooperative Wildlife Disease Study, College of Veterinary Medicine, University of Georgia, Athens, GA 30602 USA

**Keywords:** Avian paramyxovirus serotype 1, APMV-1, Exchange, Genetic diversity, Migratory bird, Newcastle disease, Order, Region, Taxa, Wild bird

## Abstract

**Background:**

Avian paramyxovirus serotype 1 (APMV-1) viruses are globally distributed, infect wild, peridomestic, and domestic birds, and sometimes lead to outbreaks of disease. Thus, the maintenance, evolution, and spread of APMV-1 viruses are relevant to avian health.

**Methods:**

In this study we sequenced the fusion gene from 58 APMV-1 isolates recovered from thirteen species of wild birds sampled throughout the USA during 2007–2014. We analyzed sequence information with previously reported data in order to assess contemporary genetic diversity and inter-taxa/inter-region exchange of APMV-1 in wild birds sampled in North America.

**Results:**

Our results suggest that wild birds maintain previously undescribed genetic diversity of APMV-1; however, such diversity is unlikely to be pathogenic to domestic poultry. Phylogenetic analyses revealed that APMV-1 diversity detected in wild birds of North America has been found in birds belonging to numerous taxonomic host orders and within hosts inhabiting multiple geographic regions suggesting some level of viral exchange. However, our results also provide statistical support for associations between phylogenetic tree topology and host taxonomic order/region of sample origin which supports restricted exchange among taxa and geographical regions of North America for some APMV-1 sub-genotypes.

**Conclusions:**

We identify previously unrecognized genetic diversity of APMV-1 in wild birds in North America which is likely a function of continued viral evolution in reservoir hosts. We did not, however, find support for the emergence or maintenance of APMV-1 strains predicted to be pathogenic to poultry in wild birds of North America outside of the order Suliformes (i.e., cormorants). Furthermore, genetic evidence suggests that ecological drivers or other mechanisms may restrict viral exchange among taxa and regions of North America. Additional and more systematic sampling for APMV-1 in North America would likely provide further inference on viral dynamics for this infectious agent in wild bird populations.

**Electronic supplementary material:**

The online version of this article (doi:10.1186/s12985-017-0714-8) contains supplementary material, which is available to authorized users.

## Background

Avian paramyxovirus serotype 1 (APMV-1) viruses are globally distributed and infect wild, peridomestic, and domestic birds. Virulent strains of APMV-1 have caused periodic epornitics of Newcastle disease in double-crested cormorants (*Phalacrocorax auritus*; order Suliformes) in North America [1–5]; been associated with outbreaks of disease in pet birds and those kept as part of zoological collections [6–8]; and are among the most economically costly poultry pathogens worldwide [9]. APMV-1 strains of low virulence and those considered to be non-pathogenic are also geographically widespread and have been detected in free-ranging populations of wild and peridomestic birds [10–13]; caged birds including animals maintained at zoos [14, 15]; and poultry reared using a wide range of production practices [16–19]. There is evidence to suggest that virulent APMV-1 strains and those of low virulence may be transmitted across wild bird-poultry interfaces [15, 20–23] and that avirulent viruses may develop increased pathogenicity in poultry [24]. Thus, the maintenance, evolution, and spread of APMV-1 viruses are relevant to the health of wild, captive, and domestic bird populations.

Despite the existence of a relatively broad body of literature on the geographic and host distribution of APMV-1 genotypes [25], much less information is available regarding viral exchange among avian species and dispersal across the landscape, particularly for viruses maintained in wild bird reservoirs. In North America, monoclonal antibody typing and sequencing of the fusion cleavage site for APMV-1 isolates derived from double-crested cormorants, a white pelican (*Pelecanus erythrorhynchos*), and a ring-billed gull (*Larus delawarensis*) associated with mortality events in Saskatchewan, Ontario, and Minnesota in 1990 and 1992 provided support for interspecies exchange of virulent APMV-1 strains among wild waterbirds and geographic spread within the mid-continent [20]. Likewise, genetically similar virulent APMV-1 strains detected in double-crested cormorants (Maine, Minnesota, Massachusetts, New Hampshire), great black-backed gulls (*Larus marinus*; Maine), a great cormorant (*Phalacrocorax carbo*; New Hampshire), and a herring gull (*Larus argentatus*; Maryland), associated with mortality events in 2010 also supports interspecies exchange and viral dispersal between Midwestern and Eastern regions of the United States of America (USA) [5]. Additionally, lentogenic APMV-1 strains, virtually genetically identical at the fusion gene, were detected among waterfowl and shorebirds sampled in Alaska during 2007 and 2009 providing evidence of viral exchange among these taxa [13]. Therefore, preliminary evidence supports the exchange of APMV-1 strains among wild waterbird taxa and dispersal among regions. However, the extent of viral exchange between wild bird taxa and among regions of North America has not been thoroughly assessed.

In this investigation, we assessed contemporary genetic diversity and evidence for inter-taxa/inter-region exchange of APMV-1 in wild birds sampled in North America. Specific objectives were: (1) to use contemporary APMV-1 isolates to identify previously unrecognized viral genetic diversity in wild birds inhabiting North America, (2) to assess viral exchange among taxonomic orders of wild birds including the potential for maintenance of viral genotypes in specific host taxa, and (3) to evaluate the dispersal of APMV-1 strains among geographic regions within North America. Results of this study provide information on viral dynamics of APMV-1 in migratory birds that may ultimately prove useful towards understanding risk associated with the emergence, exchange, and dispersal of virulent APMV-1 strains within North America.

## Methods

APMV-1 viruses were recovered via isolation in embryonated specific pathogen free eggs inoculated with swab samples collected from wild migratory birds. These isolations were opportunistic as they recovered as part of influenza A research and surveillance conducted throughout the USA during 2007–2014 [26–28]. RNA was extracted from allantoic fluid using the MagMAX AI/NDV RNA extraction kit (Ambion Inc., Austin, TX) for 58 APMV-1 isolates originating from ducks (*Anas crecca*, *A. discors*, *A. platyrhynchos*, *A. rubripes*, *A. strepera*, *Aythya americana*), gulls (*Larus argentatus, L. delawarensis*, *L. marinus*, *Leucophaeus atricilla*, *Leucophaeus pipixcan*), and shorebirds (*Arenaria interpres*, *Calidris canutus*) sampled in Delaware (*n* = 1), Louisiana (*n* = 4), Minnesota (*n* = 32), North Dakota (*n* = 3), New Jersey (n = 8), New York (*n* = 1), and Texas (*n* = 9; Additional file 1: Table S1). The complete coding region of the fusion gene was sequenced using previously described protocols [13] and primers [29]. GenBank accession numbers for complete coding sequences for fusion protein genes generated as part of this study are: KX857666–KX857723. Additionally, complete coding nucleotide sequences for fusion genes of all previously reported APMV-1 isolates were downloaded from the National Center for Biotechnology Information’s GenBank public database [30] on 26 August 2016 (*n* = 1835).

To assess contemporary genetic diversity, we applied Maximum Likelihood (ML) phylogenetic analyses to all publically available full fusion gene sequences and those obtained in this study using MEGA6 [31]. Duplicate and recombinant sequences, sequences of man-made clonal viruses, and sequences that were obtained from vaccine-like viruses were removed from the dataset in order to analyze only viruses representing natural circulation and evolution in reservoir and spill-over hosts. Preliminary pair-wise nucleotide distances were computed for all remaining fusion gene sequences, including those obtained in the current study, using MEGA6 [31] and viruses were assigned to either class I or class II per conventional classification of APMV-1 isolates. Thus, the final total of 1,483 fusion gene sequences for APMV-1 isolates was parsed into two smaller datasets representing class I (*n* = 211) and class II (*n* = 1272) sequences (Additional file 1: Table S1).

For sequences of each class I and class II, phylogenies for taxonomic assignment of genotypes/sub-genotypes were reconstructed by first assessing best-fit substitution models using MEGA6, and the goodness-of-fit for each model was measured by corrected Akaike Information Criterion (AICc) and Bayesian Information Criterion (BIC). Class I and class II trees were reconstructed using the Maximum Likelihood method based on Tamura 3-parameter and General Time Reversible models, respectively, as implemented in MEGA6 with 1000 bootstrap replicates. Estimates of average evolutionary distances among clades were inferred using the Maximum Composite Likelihood model as implemented in MEGA6 [32]. For all analyses, codon positions included were 1st + 2nd + 3rd + Noncoding and all positions containing gaps and missing data were eliminated. Fusion gene sequences were assigned to genotypes and sub-genotypes per criteria put forth by Diel et al. [33]. These criteria included average distance per site > 0.1 among genotypes and 0.03–0.1 among sub-genotypes, bootstrap support value at nodes defining genotypes/sub-genotypes > 60%, and the isolation of at least four independent viruses without a direct epidemiologic link for both genotypes and sub-genotypes.

To assess inter-taxa and inter-region exchange of APMV-1 in wild birds sampled in North America, we restricted our data to full-length fusion gene sequences for samples originating from free-ranging taxa sampled in the USA and Canada while excluding sequences from hosts which we inferred to be domestic poultry (chicken, duck, goose, turkey, and pheasant), caged or captive birds (parrots), and taxa of unknown domestic/peridomestic/captive status (i.e., hosts identified as ‘avian’ or ‘fowl’). Furthermore, we excluded sequences from environmental samples from unspecified hosts and those for which species and/or location information was incomplete. Finally, sequences obtained from pigeons were also removed from the dataset because of the synanthropic nature of these birds and a lack of information on public databases for distinguishing between wild and domestic show/race birds. Our resulting data set included 180 sequences (Table 1) for the full fusion gene of APMV-1 isolates originating from wild avian hosts of four orders (Anseriformes, Charadriiformes, Pelecaniformes, and Suliformes). Isolates were assigned to six broad geographic regions prior to further analysis: Alaska, Canada, the Western USA (West; California, Idaho, Nevada, Oregon), the Midwestern USA (Midwest; Michigan, Minnesota, North Dakota, Ohio, Wisconsin), the Gulf Coast USA (Gulf Coast; Florida, Louisiana, Texas), and the Eastern USA (East; Connecticut, Delaware, Massachusetts, Maryland, Maine, New Hampshire, New Jersey, New York, Pennsylvania). All 180 sequences were then analyzed to assess for correlations between tree topology and host taxonomic order or sample region.

**Table 1:** Distribution of APMV-1 isolates genetically characterized for this study by host order and geographic region of sample origin

Region	Anseriformes	Charadriiformes	Pelecaniformes	Suliformes	Total
Alaska	48	1	0	0	49
Canada	0	0	0	3	3
East	10	19	0	9	38
Gulf Coast	14	0	0	3	17
Midwest	45	2	1	16	64
West	2	0	0	7	9
Total	119	22	1	38	180

ML phylogenetic reconstruction of evolutionary history for North American wild bird APMV-1 isolates was conducted to evaluate consistency among ML and Bayesian approaches used throughout this study. The ML phylogenetic tree was re-constructed using Randomized Axelerated Maximum Likelihood (RAxML) v8.0.0 [34] using a general time-reversible model and gamma distributed rate variation among sites. To assess clade support, bootstrapping was performed with bootstrap convergence criterion, yielding 400 bootstrap iterations. Bayesian phylogenetic analysis was conducted using MrBayes v3.2 [35]. Two independent runs of 10 million generations each were performed using the same nucleotide evolution model described above with default priors. Bayesian Tip-association Significance testing (BaTS) [36] was then used to assess the correlation between phylogenetic topology and the character states of host order and geographic location while taking into account the uncertainty produced by phylogenetic error, using the posterior set of trees produced by our Bayesian Markov Chain Monte Carlo analysis and incorporating a 10% burn-in of each run’s chain length. For every tree in this set, BaTS calculated an Association Index (AI) [37], a Fitch parsimony score (PS) [38] and, for each trait, a monophyletic clade size statistic (MC) [36]. A p-value was produced by comparing the posterior distribution of phylogeny-trait statistics to the null distribution generated from randomized sets of taxon-character associations selected from the observed data. *P*-values ≤ 0.05 were used as evidence to support associations between tip traits (i.e., host order and geographic region of origin) and tree topology.

## Results

### Assessment of contemporary genetic diversity

**Fig. 1:**
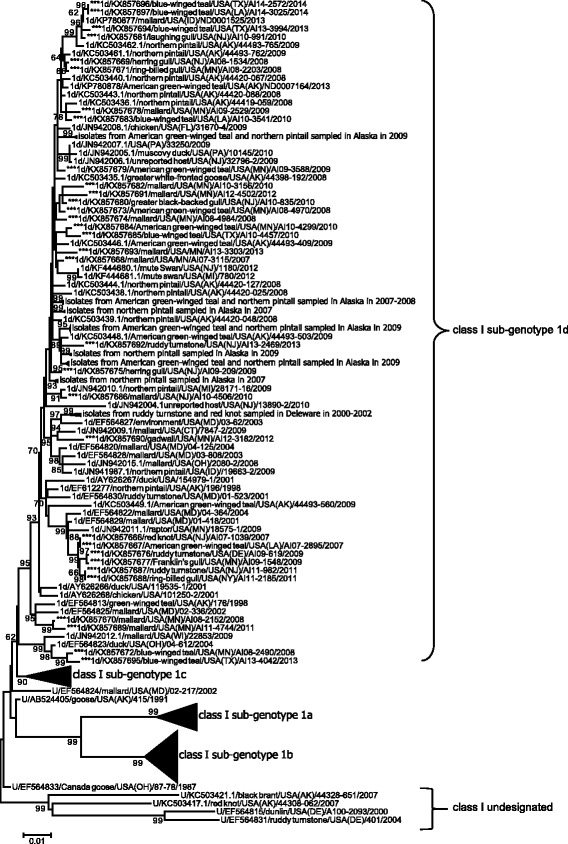
Condensed maximum likelihood phylogenetic tree depicting inferred relationship among complete nucleotide sequences for the fusion protein gene of APMV-1 class I isolates. Bootstrap support values ≥ 60 are shown to the left of branches. The tree is drawn to scale, with branch lengths measured in the number of substitutions per site. Tip labels for sequences are in the following format: taxonomic classification of isolate by sub-genotype (U = unclassified)/GenBank accession number/host/country of sample origin (and abbreviation for U.S. state if applicable)/isolate id/year of sample collection. Sequences generated for this study are indicated with asterisks (***). The complete expanded phylogeny is provided as Additional file 2: Figure S1

ML phylogenetic analyses of fusion gene sequences for APMV-1 class I isolates from wild, peridomestic, and domestic birds and 32 closely related sequences generated for this study revealed less than 0.100 average genetic differences per site among isolates, consistent with designation as a single genotype (Fig. 1; Additional file 2: Figure S1). However, four clades of sequences within class I had an average distance per site between 0.037 and 0.079 (Table 2) and bootstrap support ≥ 60 (Fig. 1; Additional file 2: Figure S1) supporting the designation of four sub-genotypes, 1a–1d, as per the nomenclature criteria for APMV-1 put forth by Diel et al. [33]. All 32 class I isolates sequenced for this study clustered within the clade classified as sub-genotype 1d (Fig. 1; Additional file 2: Figure S1). None of these 32 isolates had deduced amino acid motifs for the fusion protein cleavage site predictive of high virulence in poultry (data not shown).

**Table 2:** Average nucleotide distance between sub-genotypes for class I APMV-1 isolates

Sub-genotype	1b	1c	1d
1a	0.074	0.076	0.079
1b		0.078	0.077
1c			0.037

**Fig. 2:**
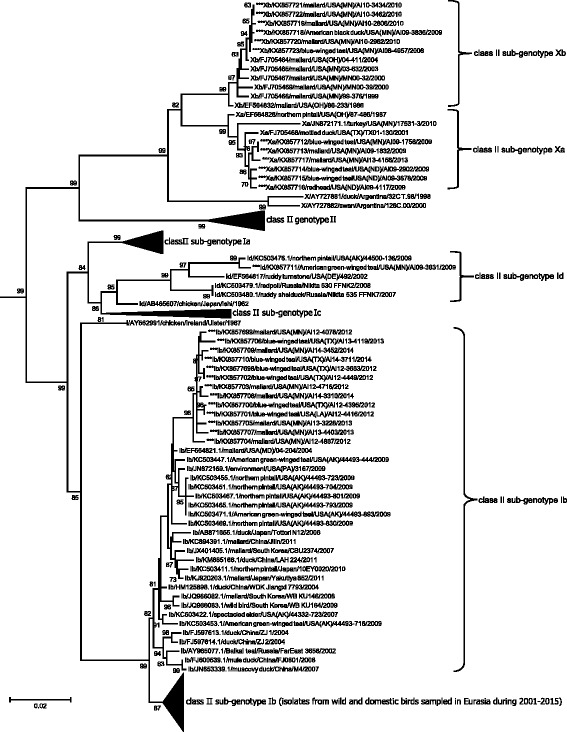
Partial condensed maximum likelihood phylogenetic tree depicting inferred relationship among complete nucleotide sequences for the fusion protein gene of APMV-1 class II genotype I, II, and X isolates. Bootstrap support values ≥ 60 are shown to the left of branches. The tree is drawn to scale, with branch lengths measured in the number of substitutions per site. Tip labels for sequences are in the following format: taxonomic classification of isolate by sub-genotype/GenBank accession number/host/country of sample origin (and abbreviation for U.S. state if applicable)/isolate id/year of sample collection. Sequences generated for this study are indicated with asterisks (***). The complete expanded phylogeny is provided as Additional file 2: Figure S2

Previously reported APMV-1 class II isolates and 26 additional closely-related sequences generated for this study clustered into seventeen clades meeting criteria for designation as distinct genotypes in ML phylogenetic analyses including an average of > 0.100 differences per site [33]. Sequences previously designated as genotype XV appeared to represent only recombinant viruses [25] and were thus excluded from the current study. Fourteen isolates sequenced for this study clustered within the clade classified as genotype I and 12 isolates sequenced for this investigation grouped with sequences in genotype X (Fig. 2; Additional file 2: Figure S2). None of these isolates clustering within genotype I or genotype X clades had deduced amino acid motifs for the fusion protein cleavage site predictive of high virulence in poultry (data not shown). Within class II genotype I, sequences clustered into four sub-clades with an average distance per site between 0.061 and 0.069 supporting the designation of four sub-genotypes (Ia–Id; Table 3). For class II genotype X, sequences clustered into two clades with an average distance per site of 0.047 supporting the designation of two sub-genotypes, Xa and Xb (Table 3).

**Table 3:** Average nucleotide distance between sub-genotypes for class II genotype I and class II genotype X APMV-1 isolates

Sub-genotype	Ib	Ic	Id	Xa	Xb
Ia	0.069	0.061	0.062	0.103	0.102
Ib		0.080	0.075	0.113	0.112
Ic			0.070	0.119	0.117
Id				0.114	0.114
Xa					0.047

### Assessment of inter-taxa/inter-region exchange

**Fig. 3:**
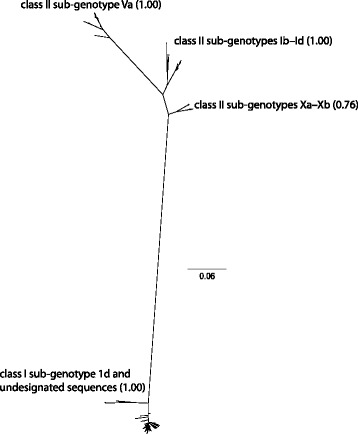
Radial Bayesian phylogenetic tree depicting the inferred genetic relationship among fusion gene sequences for 180 APMV-1 isolates originating from samples collected from wild birds in North America. Posterior probability values for major clades are shown. Dendrograms for each major clade with complete tip labels and classification of individual sequences by taxonomic order of host and North American region of sample origin are provided as Figs. 4, 5, 6 and 7

**Fig. 4:**
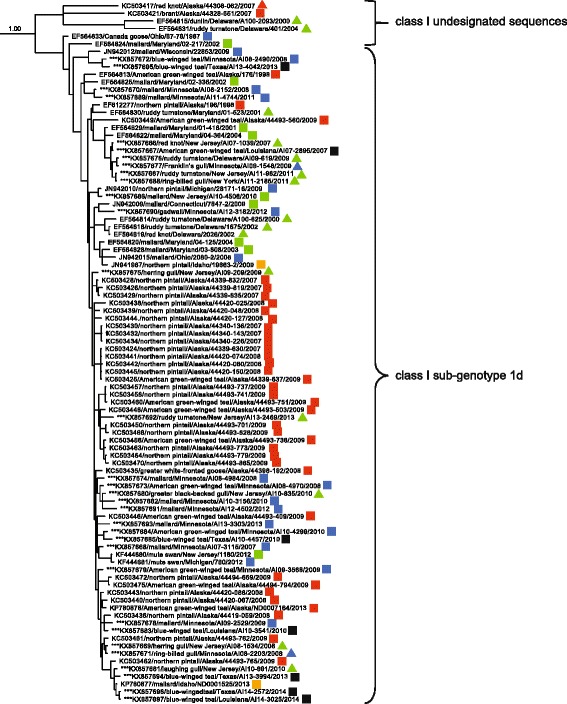
Partial Bayesian phylogenetic tree depicting the inferred genetic relationship among fusion gene sequences for 92 APMV-1 isolates originating from samples collected from wild birds in North America and defined as class I sub-genotype 1d or undesignated within class I. The Bayesian posterior probability for this major clade is indicated to the left of the first node. Sequences generated for this study are indicated with asterisks (***). Taxonomic order of host (Anseriformes = square, Charadriiformes = triangle) and region of sample collection (Alaska = red, West = orange, Midwest = blue, Gulf Coast = black, East = green) are indicated

**Fig. 5:**
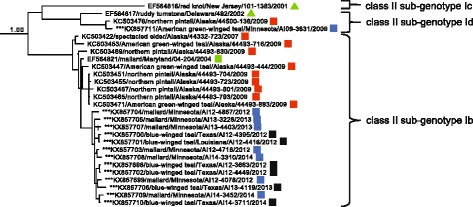
Partial Bayesian phylogenetic tree depicting the inferred genetic relationship among fusion gene sequences for 27 APMV-1 isolates originating from samples collected from wild birds in North America and defined as class II sub-genotypes Ib–Id. The Bayesian posterior probability for this major clade is indicated to the left of the first node. Sequences generated for this study are indicated with asterisks (***). Taxonomic order of host (Anseriformes = square, Charadriiformes = triangle) and region of sample collection (Alaska = red, Midwest = blue, Gulf Coast = black, East = green) are indicated

**Fig. 6:**
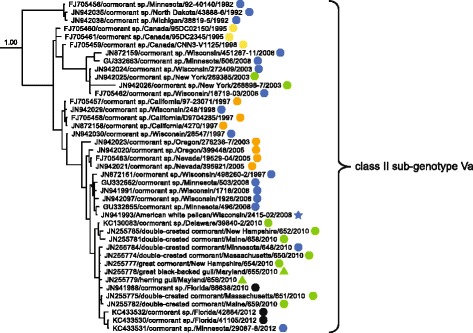
Partial Bayesian phylogenetic tree depicting the inferred genetic relationship among fusion gene sequences for 41 APMV-1 isolates originating from samples collected from wild birds in North America and defined as class II sub-genotype Va. The Bayesian posterior probability for this major clade is indicated to the left of the first node. Sequences generated for this study are indicated with asterisks (***). Taxonomic order of host (Charadriiformes = triangle, Suliformes = circle, Pelecaniformes = star) and region of sample collection (Canada = yellow, West = orange, Midwest = blue, Gulf Coast = black, East = green) are indicated

**Fig. 7:**
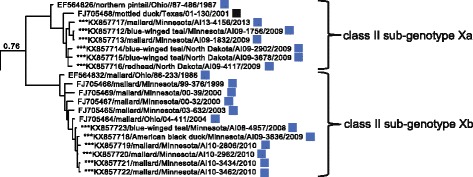
Partial Bayesian phylogenetic tree depicting the inferred genetic relationship among fusion gene sequences for 20 APMV-1 isolates originating from samples collected from wild birds in North America and defined as class II sub-genotypes Xa–Xb. The Bayesian posterior probability for this major clade is indicated to the left of the first node. Sequences generated for this study are indicated with asterisks (***). Taxonomic order of host (Anseriformes = square) and region of sample collection (Midwest = blue, Gulf Coast = black) are indicated

When we considered a sub-set of 180 fusion gene sequences originating from wild birds samples throughout North America, the ML phylogenetic tree (Additional file 2: Figure S3) depicted inferred genetic relationships consistent with ML trees to assess contemporary genetic diversity (Figs. 1 and 2) and Bayesian phylogenetic analysis used to assess associations between branch tip traits and tree topology (Figs. 3, 4, 5, 6 and 7). Fusion gene sequences for 180 wild bird origin isolates clustered into four well-supported clades (Fig. 3). These four clades corresponded to: 1. class I sub-genotype 1d and six undesignated viral sequences, 2. class II genotype I (including sub-genotypes Ib–Id), 3. class II genotype V (sub-genotype Va), and 4. class II genotype X (sub-genotypes Xa–Xb; Fig. 3). The clade comprised of sequences designated as class I sub-genotype 1d, in addition to six undesignated viral sequences, included isolates originating from waterfowl (Anseriformes) and gull/shorebird (Charadriiformes) samples collected at five of six geographic regions designated in this study (Fig. 4). Canada, the region to which the fewest APMV-1 fusion gene sequences from wild birds were assigned in this study (*n* = 3), was the only geographic location unrepresented in this clade.

The clade consisting of class II genotype I APMV-1 fusion gene sequences included viruses isolated from waterfowl (Anseriformes) and shorebird (Charadriiformes) samples originating from four geographic regions: Alaska, the Midwest, the Gulf Coast, and the East (Fig. 5). However, sequences assigned to genotype I sub-genotype Ib were exclusively from Anseriformes hosts (Fig. 5). In contrast, the clade consisting of sequences assigned to class II sub-genotype Va had differences with regard to both host and geographic region of origin. Fusion gene sequences in this clade were represented by viruses isolated primarily from cormorants (Suliformes; *n* = 38) with few sequences also originating from gulls (Charadriiformes; *n* = 2) and a pelican (Pelecaniformes; *n* = 1; Fig. 6). APMV-1 isolates clustering in this clade originated from samples collected at five of six North American regions identified in this study with a lack of sequences from Alaska (Fig. 6). Finally, the clade consisting of APMV-1 fusion gene sequences assigned to genotype X included viruses isolated exclusively from waterfowl (Anseriformes; Fig. 7). Nineteen of twenty sequences included in this clade originated from samples collected in the Midwest (Fig. 7).

When we investigated phylogenetic signals indicating limited viral transmission among divergent host taxa, trait association tests provided support for restricted APMV-1 exchange among hosts of different taxonomic orders. The AI and PS for our phylogeny both provided support (*p* < 0.01) for associations among branch tips when considering host taxa (Table 4). Furthermore, the MC provided evidence for associations between host taxonomic order for branch tips and tree topology for Anseriformes, Charadriiformes, and Suliformes (*p* ≤ 0.01; Table 4). Statistically significant associations with regard to host order were driven by monophyletic clades of APMV-1 sequences in class I sub-genotype 1d (Charadriiformes), class II sub-genotype Ib (Anseriformes), class II sub-genotype Va (Suliformes), and class II genotype X (Anseriformes).

**Table 4:** Bayesian tip-association significance testing results for APMV-1 phylogeny for wild birds isolates by host order

Statistic	Observed mean	Lower 95% CL	Upper 95% CL	Null mean	Lower 95% CL	Upper 95% CL	Significance
AI	1.80	1.31	2.30	9.20	7.92	10.32	0.00*
PS	16.46	16.00	17.00	55.92	52.60	58.93	0.00*
MC (Anseriformes)	23.00	23.00	23.00	6.56	4.26	9.65	0.01*
MC (Suliformes)	8.77	7.00	9.00	2.24	1.51	3.01	0.01*
MC (Charadriiformes)	3.93	3.00	4.00	1.57	1.00	2.08	0.01*
MC (Pelecaniformes)	1.00	1.00	1.00	1.00	1.00	1.00	1.00

Definitions of abbreviations are: AI = Association Index, PS = Fitch parsimony score, MC = monophyletic clade size statistic, and CL = confidence limit. Asterisks indicate results considered to be statistically significant (*p* ≤ 0.05)

Using a similar approach to assess relationships between the tip trait of geographic region of sample origin and Bayesian phylogenetic tree topology, we also found statistical support for restricted geographic exchange of APMV-1 within North America. Both the AI and PS metrics provided support for associations between region of sample origin for branch tips and the topology of our phylogeny (*p* < 0.01; Table 5). Additionally, MC scores provided evidence for associations between tree topology and branch tips for the geographic regions of Alaska, the West, and the Midwest (*p* ≤ 0.01; Table 5). These statistically significant associations with regard to region of sample origin were driven by monophyletic clades of APMV-1 sequences within class I sub-genotype 1d (Alaska), class II sub-genotype Ib (Alaska), class II sub-genotype Va (the West), and class II genotype X (the Midwest).

**Table 5:** Bayesian tip-association significance testing results for APMV-1 phylogeny for wild birds isolates by geographic region of sample origin

Statistic	Observed mean	Lower 95% CL	Upper 95% CL	Null mean	Lower 95% CL	Upper 95% CL	Significance
AI	6.72	6.10	7.35	13.94	12.71	15.17	0.00*
PS	54.69	53.00	57.00	96.43	92.06	101.92	0.00*
MC (Alaska)	7.03	6.00	10.00	2.55	1.97	3.51	0.01*
MC (Canada)	1.31	1.00	2.00	1.00	1.00	1.00	1.00
MC (West)	3.00	3.00	3.00	1.10	1.00	1.99	0.01*
MC (Midwest)	10.91	11.00	11.00	3.05	2.09	4.26	0.01*
MC (Gulf Coast)	2.05	2.00	2.00	1.34	1.00	2.00	0.14
MC (East)	3.21	3.00	5.00	2.10	1.47	3.01	0.12

Definitions of abbreviations are: AI = Association Index, PS = Fitch parsimony score, MC = monophyletic clade size statistic, and CL = confidence limit. Asterisks indicate results considered to be statistically significant (*p* < 0.05)

## Discussion

In this study, we found evidence to support the designation of several previously undescribed APMV-1 sub-genotypes, specifically class I sub-genotype 1d, class II sub-genotype Id, and class II sub-genotypes Xa and Xb, based on our global phylogeny of fusion gene sequences which included 58 previously unpublished wild bird origin isolates. This finding provides evidence that the full diversity of APMV-1 in wild birds has not previously been recognized and/or that this viral agent continues to evolve in natural reservoir hosts. The lack of identification of fusion gene sequences predictive of high pathogenicity in poultry from 58 previously uncharacterized wild bird APMV-1 isolates provides additional support for little or no role of the species sampled in this study (i.e., ducks, gulls, and shorebirds) in the emergence or maintenance of virulent strains of APMV-1 in North America [10, 11, 13, 39].

Inferred phylogenetic relationships among fusion gene sequences originating from wild bird samples provides evidence for exchange of APMV-1 among birds of different taxonomic orders and among geographic regions of North America; however, significant associations between tree topology and tip traits as identified through BaTS analyses also suggests that viral exchange may be greater among taxa of the same taxonomic order or within particular regions. For example, regarding the exchange of APMV-1 among taxa, entire clades of fusion gene sequences for class II sub-genotype Ib and class II genotype X were comprised exclusively of waterfowl (Anseriformes) isolates and had monophyletic clade sizes significantly larger than 95% confidence intervals for what would be expected by chance. This suggests that this viral diversity may have evolved and been maintained exclusively in waterfowl species. Alternatively, unidentified sampling and diagnostic biases may have led to this result. We acknowledge that the species represented in this study were limited and may not reflect the full host range of APMV-1 lineages.

In contrast to the finding of monophyletic clades of sequences from Anseriformes hosts, the large clade comprised of class I sub-genotype 1d sequences contained numerous isolates originating from both waterfowl and gulls/shorebirds (Charadriiformes) with smaller monophyletic sub-clades of waterfowl sequences. Thus, there is evidence to suggest that class I sub-genotype 1d APMV-1 may be readily shared among Anseriformes and Charadriiformes hosts. There were, however, two small monophyletic sub-clades of Charadriiformes sequences within the greater class I sub-genotype 1d clade which were significantly larger than expected by chance. As these gull/shorebird isolates were derived from samples collected in different regions and/or years, it is plausible that viral diversity is occasionally maintained exclusively within Charadriiformes hosts and then infrequently exchanged with sympatric waterfowl hosts. Additional and more systematic sampling for APMV-1 in Anseriformes and Charadriiformes hosts would be beneficial for improving inference on the viral dynamics between these taxonomic groups.

Similar to evidence for restricted exchange of APMV-1 observed for a sub-set of the viral diversity detected in waterfowl, the clade of sequences designated as class II sub-genotype Va was comprised nearly exclusively of sequences from cormorants (Suliformes) with the exception of two sequences originating from gulls (Charadriiformes) and a single viral sequence from a pelican (Pelecaniformes). As such, the mean monophyletic clade of cormorant APMV-1 fusion gene sequences within the class II sub-genotype Va clade exceeded the upper 95% confident limit expected by chance and provides support the for the evolution and maintenance of such viruses by cormorants. Other infected hosts, such as gulls and pelicans, are likely the result of spill-over infections. We caution, however, that as stated previously for other APMV-1 lineages, the possibility that unidentified sampling and diagnostic biases affected our results cannot be dismissed.

With regard to the exchange of APMV-1 among regions, clades for all four genotypes in our phylogeny for North American wild bird isolates were comprised of sequences originating from more than one region which suggests some degree of geographic dispersal of this viral agent by migratory birds. However, significant associations of branch tips for three regions within our phylogeny also provides evidence for either epidemiologic linkage of viruses analyzed in this study (e.g., viruses related to a single outbreak) or unrecognized mechanisms in hosts or the environment that have resulted in restrictions in the geographic extent of viral exchange for sub-sets of the genetic diversity of APMV-1. For example, statistical support for associations between region of sample origin and tree topology was observed for Alaska and the West. These results were driven by monophyletic clades of APMV-1 sequences for viruses isolated from waterfowl (Anseriformes) in Alaska (class I sub-genotype 1d and class II sub-genotype Ib) and cormorants (Suliformes) in the West (genotype Va). All of these monophyletic clades represent sequences derived from isolates recovered from samples originating within the same region within a span of three years. Thus, statistical support for associations of tip traits within these clades may be best explained by exchange of viruses among sympatric taxa or infection from a common source during localized outbreaks rather than biotic or abiotic limits to viral dispersal into or out of Alaska and the West.

Stronger evidence for biologically significant geographic associations of branch tip traits and tree topology for APMV-1 was evidenced by the genetic relationship among sequences designated as class II genotype X. Nineteen of 20 sequences assigned to this genotype originated from waterfowl in the Midwest with the single exception being an isolate recovered from a mottled duck (*Anas fulvigula*) in Texas. As genotype X isolates sequenced for this study were recovered from samples collected from 1986–2013, a temporal period spanning 27 years, it is unlikely epidemiologic linkage related to a single outbreak can completely explain this result. Although genotype X isolates have also previously been detected in waterfowl in Argentina, isolates from North America are phylogenetically distinct from those detected in South America (Fig. 2; Additional file 2: Figure S2). Thus, there may be biotic or abiotic limitations restricting the circulation of APMV-1 class II genotype Xa–Xb viruses beyond waterfowl inhabiting the Midwest that warrant additional investigation. Alternatively there may be cryptic epidemiological influences in our sampling approach that influenced our results (e.g., peak in prevalence of genotype X viruses coincident with sampling events in the Midwest or an unsampled reservoir host for such viruses with restricted range).

## Conclusion

In summary, through analysis of APMV-1 fusion gene sequences for isolates recovered from wild birds sampled in North America, we found evidence for previously unrecognized viral diversity circulating among ducks, gulls, and shorebirds. We did not, however, find any evidence that such diversity was likely to be pathogenic to poultry or that this diversity may contribute to the emergence of avian pathogens in North America. When we assessed the exchange of APMV-1 among taxa, we found evidence for incomplete restrictions in viral sharing among taxa for some viral lineages (e.g., class II sub-genotypes Ib, Va, Xa, and Xb) whereas other viral diversity appeared to be more readily exchanged among taxa (e.g., class I sub-genotype 1d). Finally, while our results support incomplete restrictions with regard to geographic dispersal by migratory birds for some APMV-1 viral lineages, this result was likely influenced by epidemiologic linkage. However, the association between class II genotype X viruses and waterfowl in the Midwest warrants further investigation.

## Additional files

Additional file 1: Table S1. APMV-1 complete fusion protein gene sequences used for constructing maximum likelihood phylogenetic tree for assessment of genetic diversity. Sequences generated as part of this study are indicated in bold font. (XLSX 89 kb)
Additional file 2: Figure S1. Maximum Likelihood phylogeny depicting the inferred relationship among fusion gene sequences for APMV-1 class I isolates available on The National Center for Biotechnology Information GenBank as of 26 August 2016 (*n* = 211). The tree with the highest log likelihood (−14761.8677) is shown. Bootstrap support values ≥ 60 are shown. The tree is drawn to scale, with branch lengths depicting the number of substitutions per site. All positions containing gaps and missing data were eliminated. There were a total of 1662 positions in the final dataset. Branch tips include sub-genotypes assigned to major clades meeting criteria described by Diel et al. (2012; *U* = unclassified), followed by the GenBank accession number, host name, country of isolation, strain designation and year of isolation (if available). Sequences generated for this study are indicated with asterisks (***). **Figure S2.** Maximum Likelihood phylogeny depicting the inferred relationship among fusion gene sequences for APMV-1 class II isolates available on The National Center for Biotechnology Information GenBank as of 26 August 2016 (*n* = 1272). The tree with the highest log likelihood (−82565.4161) is shown. Bootstrap support values > 60 are shown. The tree is drawn to scale, with branch lengths depicting the number of substitutions per site. All positions containing gaps and missing data were eliminated. There were a total of 1644 positions in the final dataset. Branch tips include sub-genotypes assigned to major clades meeting criteria described by Diel et al. (2012; *U* = unclassified), followed by the GenBank accession number, host name, country of isolation, strain designation and year of isolation (if available). Sequences generated for this study are indicated with asterisks (***). **Figure S3.** Maximum Likelihood phylogenetic tree depicting the inferred genetic relationship among fusion gene sequences for 180 APMV-1 isolates originating from samples collected from wild birds in North America. Bootstrap support values for major clades are shown. Sequences generated for this study are indicated with asterisks (***). Taxonomic order of host (Anseriformes = square, Charadriiformes = triangle, Suliformes = circle, Pelecaniformes = star) and region of sample collection (Alaska = red, Canada = yellow, West = orange, Midwest = blue, Gulf Coast = black, East = green) are indicated. (PDF 1177 kb)

## Abbreviations

AI: Association Index
APMV-1: Avian paramyxovirus serotype 1
MC: Monophyletic clade size statistic
PS: Fitch parsimony score
RNA: Ribonucleic acid
USA: United States of America
