# Supplementary figures and images for: Assessment of contemporary genetic diversity and inter-taxa/inter-region exchange of avian paramyxovirus serotype 1 in wild birds sampled in North America
Source: Virol J. 2017 Mar 3;14:43. doi: 10.1186/s12985-017-0714-8 (PMC5335501; doi:10.1186/s12985-017-0714-8)

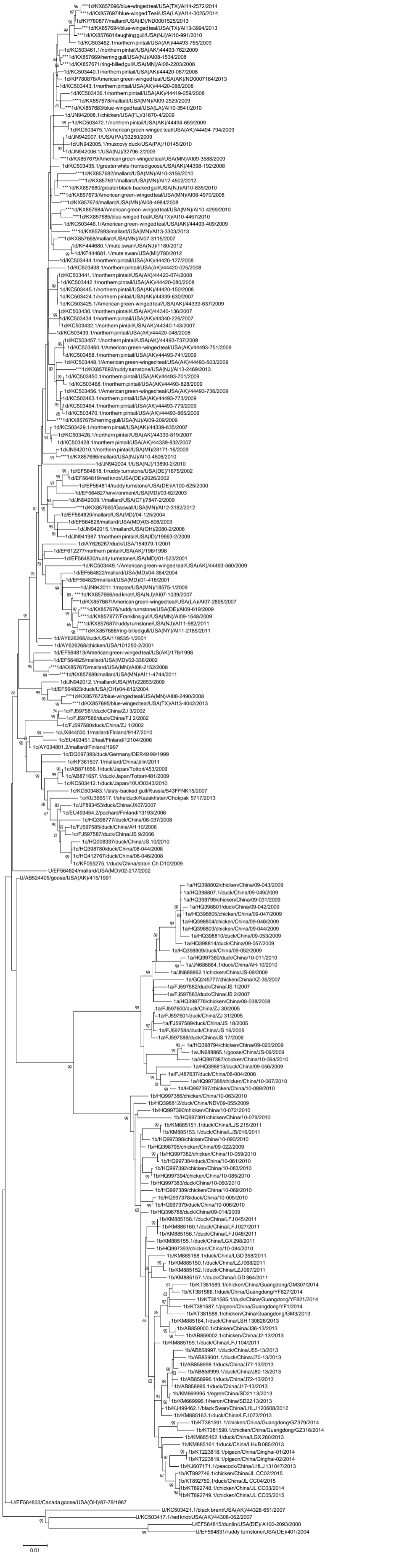

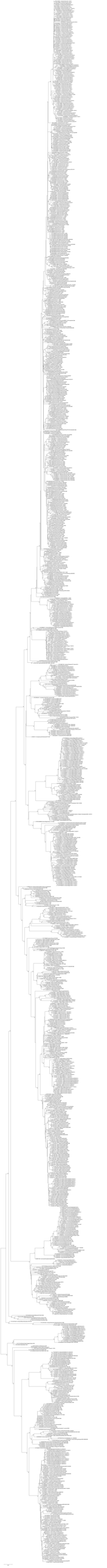

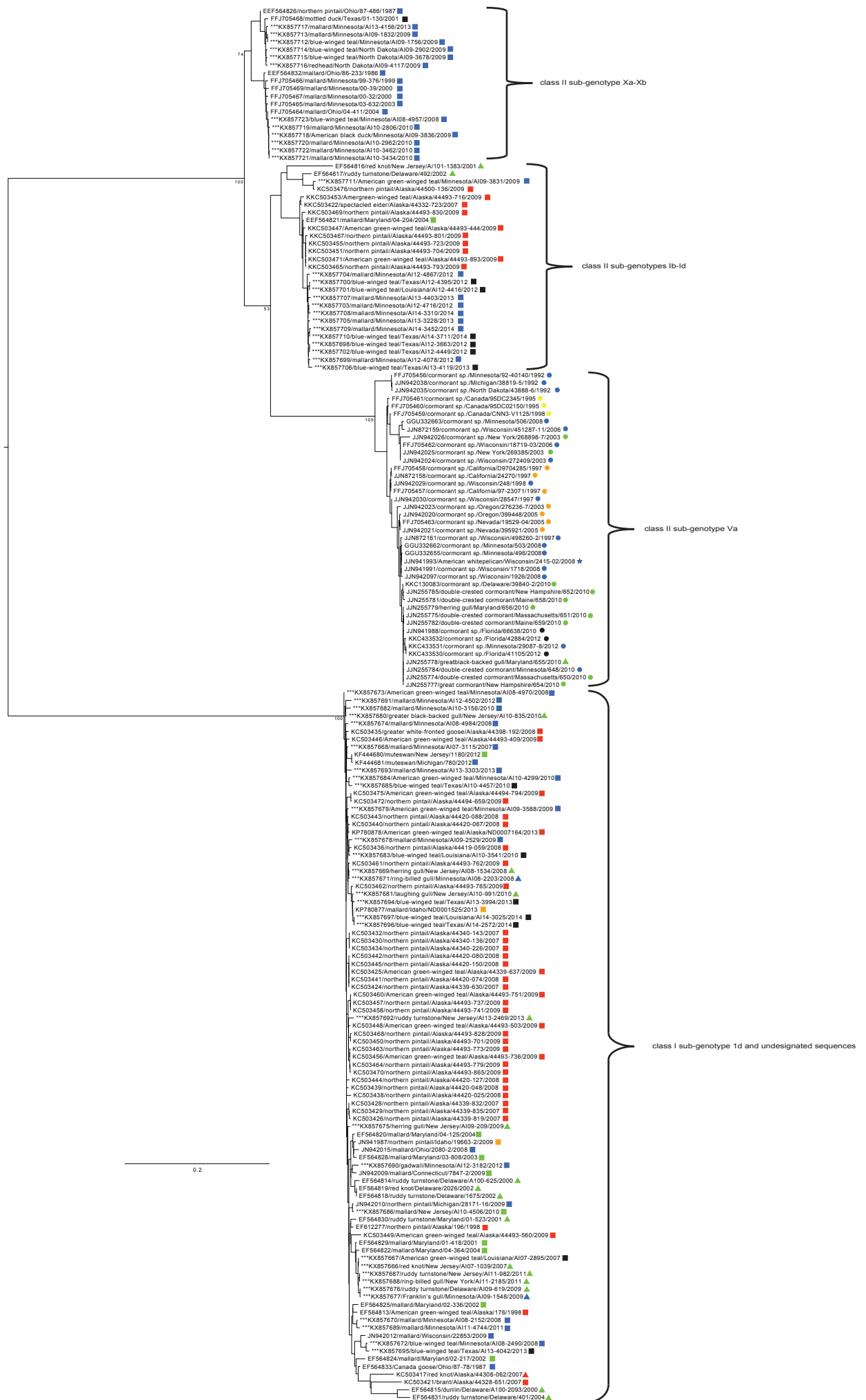

Supplement: Additional file 2: Figure S1. — Maximum Likelihood phylogeny depicting the inferred relationship among fusion gene sequences for APMV-1 class I isolates available on The National Center for Biotechnology Information GenBank as of 26 August 2016 (n = 211). The tree with the highest log likelihood (−14761.8677) is shown. Bootstrap support values ≥ 60 are shown. The tree is drawn to scale, with branch lengths depicting the number of substitutions per site. All positions containing gaps and missing data were eliminated. There were a total of 1662 positions in the final dataset. Branch tips include sub-genotypes assigned to major clades meeting criteria described by Diel et al. (2012; U = unclassified), followed by the GenBank accession number, host name, country of isolation, strain designation and year of isolation (if available). Sequences generated for this study are indicated with asterisks (***). Figure S2. Maximum Likelihood phylogeny depicting the inferred relationship among fusion gene sequences for APMV-1 class II isolates available on The National Center for Biotechnology Information GenBank as of 26 August 2016 (n = 1272). The tree with the highest log likelihood (−82565.4161) is shown. Bootstrap support values > 60 are shown. The tree is drawn to scale, with branch lengths depicting the number of substitutions per site. All positions containing gaps and missing data were eliminated. There were a total of 1644 positions in the final dataset. Branch tips include sub-genotypes assigned to major clades meeting criteria described by Diel et al. (2012; U = unclassified), followed by the GenBank accession number, host name, country of isolation, strain designation and year of isolation (if available). Sequences generated for this study are indicated with asterisks (***). Figure S3. Maximum Likelihood phylogenetic tree depicting the inferred genetic relationship among fusion gene sequences for 180 APMV-1 isolates originating from samples collected from wild birds in North America. Bootstrap suppor [file 12985_2017_714_MOESM2_ESM.pdf]
